# Many diseases, one model of care?

**DOI:** 10.15256/joc.2016.6.73

**Published:** 2016-02-17

**Authors:** Tit Albreht, Mariana Dyakova, François G. Schellevis, Stephan Van den Broucke

**Affiliations:** ^1^Centre for Health System Analyses, National Institute of Public Health, Ljubljana, Slovenia; and CANCON Joint Action Programme, Ljubljana, Slovenia; ^2^Health Sciences, Warwick Medical School, University of Warwick, Coventry, UK; ^3^NIVEL (Netherlands Institute for Health Services Research), Utrecht, The Netherlands; and Department of General Practice and Elderly Care Medicine/EMGO Institute for Health and Care Research, VU University Medical Center, Amsterdam, The Netherlands; ^4^Psychological Sciences Research Institute, Université catholique de Louvain, Louvain-la-Neuve, Belgium

**Keywords:** Multimorbidity, European Partnership for Action Against Cancer (EPAAC), Innovating Care for People with Chronic Conditions in Europe (ICARE4EU) project, Cancer Control Joint Action (CanCon), diabetes, literacy, multiple chronic conditions, comorbidity

## Abstract

Patients with multiple chronic conditions (multimorbidity) have complex and extensive health and social care needs that are not well served by current silo-based models of care. A lack of integration between care providers often leads to fragmented, incomplete, and ineffective care, leaving many patients overwhelmed and unable to navigate their way towards better health outcomes. In planning for the future, healthcare policies and models of care are required that cater for the complex needs of patients with multimorbidity and that deliver coordinated care that is patient-centred and focused on disease prevention, multidisciplinary teamwork and shared decision-making, and on empowering patients to self-manage. Salient lessons can be learnt from the work undertaken at a European and national level to develop care models in cancer and diabetes – two complex and often co-occurring conditions requiring coordinated long-term care. Innovative work is also underway in many European countries aimed at improving the integration of care for people with multimorbidity, resulting in more efficient and cost-effective health outcomes. This article reviews some of the most innovative programmes that have been initiated across and within Europe with the aim of improving the way care is delivered to people with complex and multiple long-term conditions. This work provides a foundation upon which to build better, more effective models of care for people with multimorbidity.

## Introduction

It has been estimated that 50 million people in Europe are living with multiple chronic conditions (multimorbidity), and as the population grows and ages, this number will increase further, especially amongst those of working age and the elderly. Current health and social care structures in Europe are predominantly disease-orientated, with models and organizations of care built around silos of expertise in a single medical condition. Patients with multimorbidity have complex and continuous health and social care needs requiring monitoring and intervention from various professionals who focus on the individual rather than on the conditions. This poses major challenges in terms of care coordination, coherence, funding, safety, and optimization of health outcomes, as well as increasing health literacy and self-care capability of patients and their families. 

In planning for the future, health and social policies and models of care must be developed in Europe that cater for the complex needs of patients with multimorbidity, delivering care that is both effective and sustainable. Much work has already been undertaken across Europe in developing care models for chronic and complex medical conditions, such as cancer and diabetes, and there is progress towards more integrated care for patients with multimorbidity. This article reviews some of the most innovative programmes that have been initiated across Europe with the aim of improving the way care is delivered to people with complex and multiple long-term conditions.

## Integrated care: what can we learn from models of care developed for cancer?

Continued medical advances and enhanced healthcare systems have led to the transformation of many previously life-threatening, acute medical conditions into chronic, life-long illnesses that require ongoing care. Today, this is as true for some cancers as it was decades ago for people with communicable diseases, such as tuberculosis, human immunodeficiency virus infection, and acquired immune deficiency disease or, on the other hand, non-communicable diseases, such as ischaemic heart disease or diabetes. Improved cancer survival rates and a broader understanding of how healthcare systems can contribute to improving the health of populations has led to an evolution of cancer care from one of disease-focused management to a more patient-centred approach in which greater attention is paid to psychosocial morbidities, quality of life, patient empowerment, and survivorship. Cancer is predominantly an age-related condition, with the number of cases predicted to increase as the European population ages and survival improves further. This brings with it a wide range of challenges, not least of which is the issue of multimorbidity and how this is managed within cancer care models.

### European Partnership for Action Against Cancer (EPAAC)

Two major European initiatives have been launched in recent years to encourage European governments to address the multiple cancer-related challenges within their own healthcare systems. The EPAAC (www.epaac.eu) was launched in 2009, under the umbrella of the European Commission, with the initial goal of characterizing and building upon the knowledge and expertise captured within existing national cancer control policies [1]. A second early objective of the EPAAC was to satisfy the need for an integrated and structured approach to tackling cancer in each member state, with teams of experts addressing issues including health promotion and prevention, screening and early diagnosis, research, information and data, and national cancer plans [1]. In April 2014, after extensive collaboration, research, and debate, the EPAAC published its “European guide for quality national cancer control programmes” [2], containing a synthesized description of the broad range of cancer control services that may be offered through national health systems and a proposed list of indicators that countries may consider in order to improve the monitoring and evaluation of their plans. A key goal of the guide was to encourage convergence in national approaches to cancer control programme planning, with the aim of fostering the ability of policy analysts to compare plans within, and across, European borders, and to support a common understanding of cancer planning among policy makers in the European Union (EU) [2]. Although the guide does not specifically address the issue of multimorbidity in cancer care, several relevant sections can be highlighted. 

Cancer prevention strategies outlined in the guide, which include cancer risk assessments, the development of national policies, and legislation to tackle modifiable risk factors (e.g. tobacco smoking, alcohol intake, diet, physical activity, and obesity), would positively impact many chronic conditions affecting the elderly and other high-risk groups. There seems little doubt that, as the pensionable age increases and more people work into their late 60s and 70s, prevention of cancer and other long-term conditions will become increasingly important from both an individual and a societal perspective. The critical role of primary care teams in health promotion and the prevention of cancer, and in the ongoing management of cancer survivors, is emphasized in the guide, and this is highly relevant to the management of multimorbidity. Many experts believe that primary care teams should have a more prominent role in cancer care; however, in many European countries, primary care is not the point of healthcare entry for patients, and these patients remain under specialist care even after their initial treatment has been completed. Whichever model of care is applied, whether it is primary- or secondary-care driven, the key issue for cancer patients and all those with complex medical needs, is that their care pathway is clear, readily accessible, and organized to support all aspects of their condition(s), including psychosocial morbidities, activities of daily living, social care needs, and survivorship issues. 

### Cancer Control Joint Action (CanCon)

A more recent EU-wide initiative, CanCon (www.cancercontrol.eu), which is co-funded by participating organizations and the Health Programme of the EU, was launched in 2014 and will continue until 2017. As with the EPAAC, the ultimate goal of CanCon is to contribute towards reducing the cancer burden in the EU and improving the quality of care among member states. To achieve this, the Joint Action group, which includes hundreds of cancer experts, is working on plans to improve overall cancer control through quality-based cancer screening programmes, better integration of cancer care, community-based cancer care approaches, and the development of a European organizational framework that addresses issues of survivorship and rehabilitation [3]. These elements will be combined with other relevant aspects of cancer control to form a European guide on quality improvement in comprehensive cancer control, with the final report due to be published in March 2017 [3]. 

Optimal decision-making in the diagnosis, treatment, and support of cancer patients increasingly involves multidisciplinary teams, with a growing number of specialists and healthcare professionals providing a broader range of interventions addressing different aspects of cancer care [4]. At a European level, health systems can benefit from taking a multidisciplinary approach to care planning, with comprehensive care pathways assisting patients and professionals to navigate often complex health systems [4]. The same approach could readily be applied to the management of other chronic conditions, comorbidities, and multimorbidities, contributing to a smoother process, more efficient management of these conditions and, ultimately, better clinical outcomes. 

## Self-management: what can we learn from the diabetes experience?

Patient empowerment through self-management education is key to the treatment of chronic disease, such as type 2 diabetes, and to the prevention of complications. Patients with diabetes often have multimorbidity and can therefore be considered as models through which to evaluate the role of self-management in multimorbidity care. As the burden of diabetes continues to grow throughout Europe, many EU member states have put in place national diabetes plans or policy frameworks. The most successful of these frameworks addresses both psychosocial and medical needs and encourages patient autonomy through disease education and self-management [5]. Diabetes self-management education has proven health benefits [6, 7], and is widely recommended. However, questions remain with regard to its cost-effectiveness, the best method of delivery, and the impact of patient and provider characteristics on self-management education effectiveness. To address some of these questions, the European Diabetes Literacy Project (www.diabetesliteracy.eu) was initiated in 2012 with financial support from the European Commission under its FP7 programme. The aim of the project was to assess the cost and effectiveness of different forms of self-management education in diabetes, and to investigate potential moderators of effectiveness, notably the patient’s level of health literacy, the organizational characteristics of the setting in which the education is offered, and the implementation fidelity, with a view to increase the effectiveness of diabetes self-management education in EU member states as part of an EU-wide comprehensive diabetes strategy [8]. Partners from seven EU member states (Belgium, Denmark, Germany, Ireland, Austria, the Netherlands and the UK), as well as from Israel, the USA and Taiwan, contributed to the project, undertaking a comprehensive analysis of national diabetes strategies and diabetes self-management education programmes, evaluating their effectiveness, cost-effectiveness, and influencing factors [8]. The results of this work will be published in full in the beginning of 2016.

Several key messages have arisen from this work that can be applied to the care of patients with multimorbidity. Firstly, and most importantly, there is evidence of a growing acceptance that self-management education is a core component of diabetes care, with many European countries (but not yet all), incorporating self-management into their national programmes. Secondly, many different types of self-management education programmes already exist in Europe and in the other partner countries studied, providing a solid foundation upon which to build in the future. No evidence was found to suggest that any one approach to self-management education was significantly more effective than another, which implies that relatively cheaper forms of self-management education, such as group programmes, are as effective as one-to-one education. An interesting finding was also that self-management education was effective even when providers did not strictly adhere to the programme guidelines. In fact, adaptation of the programme by the trainers was sometimes associated with a greater improvement, particularly when the changes concerned the coverage rather than the content of the programme. On the other hand, it was also seen that self-education programmes do not always reach the patients who are most in need. Moreover, there seems to be an under-representation of peer-led self-management support and education, as well as an underdevelopment of IT-based programmes, suggesting that more work is needed in these areas. 

The cost of diabetes self-management education varied widely between countries and between the different types of programmes operating within countries. However, compared with the overall cost of diabetes care, the cost of these programmes is relatively low, with the more expensive approaches costing approximately €10–15 per patient per hour. Since most programmes take at least 10 sessions to create positive health benefits, at a total cost of €100–150 per patient, diabetes self-management education can be considered a cost-effective intervention.

Given the availability of existing self-management education, a key recommendation resulting from this project is that, rather than investing in the development of new programmes, existing ones should be made available to more patients, but tailored towards the cultural, demographic, and health literacy characteristics of the participants. Moreover, patients should be involved in the planning and adaptation of existing educational approaches. In addition, a broader range of professionals could be involved in self-management education, and the training of these professionals could be intensified and improved, with a greater focus on behavioural and psychosocial aspects of diabetes care. Further consideration could also be given to developing web-based diabetes education programmes for people with low health literacy levels. The study showed that it is possible to develop IT-based programmes that engage people with lower health literacy, but are also acceptable for people with higher levels. Finally, and crucially, the efficiency and cost-effectiveness of diabetes self-management education must be rigorously evaluated on an ongoing basis in order to expand the evidence base supporting its use.

## Managing multimorbidity: what can we learn from the Innovating Care for People with Multiple Chronic Conditions in Europe (ICARE4EU) project?

Multimorbidity has a profound impact on the lives of affected individuals in terms of their physical, psychological, and social well-being. Multimorbidity also presents a major challenge for healthcare systems, with complex medical needs requiring input from multiple providers both within and outside healthcare settings. Current healthcare systems are typically built around individual medical specialties, and a lack of co-ordination across these specialities often leads to fragmented, incomplete, and ineffective care for patients with multimorbidity. Integrated care programmes, which are patient-centred, proactive, and provide well-coordinated multidisciplinary care, are increasingly recognized as being more effective care models for people with complex medical needs, and are most likely to benefit people with multimorbidity. 

### Innovating care for people with the ICARE4EU project

To explore this issue further and to assess current practices in terms of integrated care for people with multimorbidity in Europe, the ICARE4EU project (www.icare4eu.org) was initiated in 2013 with financial support from the Health Programme 2008–2013 of the European Commission. The project approached country experts in 31 European countries, requesting information on existing integrated care programmes for people with multimorbidity in each country, including their strengths and weaknesses, inputs, processes, and outcomes [9]. One of the most alarming findings from this preliminary work was that there were almost no national integrated care policies directed at the management of patients with multimorbidity [10, 11]. Regional policies existed in some areas of Italy; and in Germany, Italy, and the Netherlands, condition-specific policies focusing on chronic illnesses were identified [10].

In contrast to the lack of national or regional policies for the integrated management of multimorbidity, 101 different innovative approaches aimed at improving the care of people with multimorbidity through integrated programmes were identified in 24 European countries [11]. Most of these approaches were operating at a local or regional level, with the largest numbers of initiatives identified in Spain (*n*=15), Greece (*n*=9), and Germany (*n*=8). No programmes that met the inclusion criteria (see Appendix A1 [11]) were found in France, Romania, Czech Republic, Hungary, Poland, Slovakia, or Estonia. 

According to the literature, the primary aim of integrated care is to reduce fragmentation of care and costs in order to improve clinical outcomes, quality of life, patient satisfaction, effectiveness, and efficiency [12]. According to the ICARE4EU study, the main objectives of the 101 integrated care programmes addressing multimorbidity included improving access to care, improving the quality of care, improving patient centredness and patient outcomes, and optimizing care utilization and costs [11]. In 80% of programmes identified, one of the main objectives was to increase the level of multidisciplinary collaboration. Improving patient involvement (71%), improving the coordination of care (71%), and reducing hospital admissions (69%) were also listed as key objectives. Most of the programmes focused on multimorbidity in general (59%), while some addressed a specific diagnosis with a range of comorbidities (28%), and a few tackled a combination of specific diagnoses (14%) [11].

**Figure 1:**
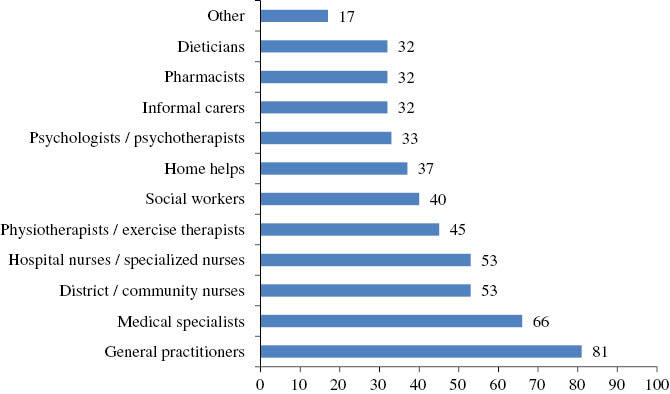
Percentage of care providers involved in selected integrated care programmes directed at multimorbidity (*n*=101) [11]. Reproduced from van der Heide I, *et al.* Innovating care for people with multiple chronic conditions in Europe: an overview. Utrecht: NIVEL; 2015. Available from: http://www.icare4eu.org/pdf/State-of-the-Art_report_ICARE4EU.pdf

Various organizations and stakeholders were involved in the 101 integrated care programmes selected. Almost two-thirds of the programmes involved primary care practices (70%) and more than one-half involved general hospitals (57%). The initiating organization was frequently a government body (in 40% of programmes), a hospital (24%), or a primary care organization (34%). The care providers most frequently involved in the programmes were general practitioners (Fig. 1) [11].

Overall, the quality of these programmes was considered to be excellent, and there was a strong potential to upscale many of them for wider implementation. Of the 101 programmes identified, eight were selected for a more in-depth analysis (Table 1) [13–20], and further details of these are available on the ICARE4EU website (www.icare4eu.org). 

#### Belgium

In Belgium, the “PROTOCOL 3” programme (an agreement between the Federal State, regions, and communities) has been initiated to develop alternative models of care for the frail elderly, with the aim of delaying or preventing institutionalization [13]. This is a good example of a nationally supported programme, which initially funded 63 projects, with a further 26 projects supported in a second round of research grant awards. The first 63 projects have now been scientifically evaluated by a consortium of Belgian universities, which concluded that, overall, the programme had been cost-effective in terms of reducing the risk of institutionalization.

#### Bulgaria

In Bulgaria, a programme founded by a regional non-profit organization to provide diabetic care was selected from the 101 programmes to illustrate good practice in terms of helping patients with diabetes and comorbidity to navigate the secondary care system and empowering and educating them to improve their self-management skills [14]. The programme is primarily targeted at patients with diabetes and their families, with the aim of providing access to care regardless of frailty, age, or insurance status. All healthcare providers within the programme work on a voluntary basis, with funding from donations, member’s contribution fees, and a small annual contribution from the local government. The programme continues to extend its scope and network and has already been transferred to other neighbouring regions. 

#### Cyprus

An advanced telemedicine programme (called the TeleRehabilitation programme) has been initiated in Cyprus, illustrating how telehealth can be used to overcome geographical inequality [15]. The programme encompasses a home-based rehabilitation service that applies advanced telemedicine to patients who have been discharged from an intensive care unit (ICU) and may have financial and/or mobility-related issues preventing them from attending in-hospital rehabilitation services. Patients are provided with a computer and touch-screen monitor at home that enables them to interact with a physiotherapist remotely, with vital signs monitored using a portable device connected via the Internet. The programme has improved adherence to rehabilitation, improved the health status of patients, and reduced the risk of readmission to an ICU. It has also proved to be cost-effective with high levels of patient satisfaction.

#### Denmark

In Denmark, the Clinic for Multimorbidity and Polypharmacy (CMP) – the Diagnostic Centre of Silkeborg Regional Hospital in Silkeborg – has developed a unique, holistic approach to managing patients with multimorbidity [16]. The clinic provides a comprehensive, multidisciplinary approach to care by integrating all relevant members of the healthcare team, including a pathway coordinator, the medical doctor, nurse, pharmacist, physiotherapist, occupational therapist, psychiatrist, and other relevant specialists, in a 1-day assessment of the patient. This same-day service includes a thorough evaluation of the patient’s disease status by the healthcare team, as well as a review of their medication plan and follow-up recommendations. The integrated care model has resulted in more efficient and effective use of resources, more effective knowledge sharing, and increased treatment quality and patient satisfaction. 

#### Finland

In Finland, the Putting the Patient in the Driver’s Seat (POTKU) project is an excellent example of patient-centredness and integration, especially across primary care [17]. The project was targeted at people with a chronic condition seeking care from a local primary care health centre, with many of these patients having multimorbidity. The programme used a modified version of the Chronic Care Model [21] as its theoretical framework, with personal health and care plans developed for 16,000 people during the project. Clientship profiles were used to assess the patients’ self-management skills, and a pathway of care for people with multimorbidity was developed to integrate care services and to improve patient-centred cooperation amongst care professionals. The programme illustrates the potential benefits of developing personalized care plans and care pathways, and supporting self-management and patient education when faced with multimorbidity. Unfortunately, funding for the programme has ceased; however, some elements of the programme are now structurally embedded in the care process within health centres.

#### Germany

The German Gesundes Kinzigtal programme [18] provides a good example of financial innovation, and uses a novel business model that could potentially be applied across Europe. The overall aim of the programme was to invest in disease prevention and to manage care processes more intelligently in order to maintain health, improve the quality of life of patients, and avoid unnecessary costs. The programme involved the entire population of one region in Germany who was insured by two sickness funds, and specific models of care were developed for people with multimorbidity based on the Minimally Disruptive Medicine care model [22]. Individuals with multimorbidity were, or will be, offered specific interventions including medication optimization reviews to address polypharmacy and self-management training. Physicians will receive “digital cockpit reports” to help them assess and compare their own prescribing behaviour. Other key elements of the programme include the development of a more patient-centred approach to treatment planning, improved coordination of care, and the wider use of e-Health. The financial goal of the programme has been to invest in the health gain of its members in order to result in cost savings – a goal that has been achieved. Overall, in the 7 years since its launch, the programme has led to a paradigm shift in the way healthcare is delivered in the region, resulting in better health outcomes, shared decision-making, and substantial cost savings.

#### Spain

The Strategy for Chronic Care in Valencia Region (Estrategia para la atención a paceientes crónicos en la Comunitat Valenciana), Spain, is an innovative approach that has been established to provide integrated care for people with multimorbidity in Spain [19]. The care model is unique in that is constructed around the patient, with integration between hospital, primary, and community health services. Case managers are assigned to continuously monitor the patients, thereby ensuring continuity and quality of the care process. The model considers the patients’ own wishes and needs and provides appropriate, customized support to them, their carers, and their family. Information and communication technologies, as well as decision support systems, are also provided for stratifying the population by risk and for monitoring and rationalizing drug therapies. To date, the integrated care model has been successfully implemented, with benefits to patients, healthcare professionals, and the health sector. Preliminary findings suggest significant cost savings in terms of public expenditure on drugs. 

#### The Netherlands

The Integrated Care (INCA) model being developed in the Netherlands is focusing on providing integrated care for people with multimorbidity [20], and is an example of a well-planned, comprehensive national model. A key element of the programme is to translate existing Dutch care standards and protocols into an integrated modular approach to care focusing on lifestyle, medical interventions, and psychosocial aspects. Individual care plans are developed with the patient on the basis of a risk profile and the patient’s personal perspective of their health and lifestyle issues. Several stepped-care modules have already been developed for cardiovascular disease, type 2 diabetes, and chronic obstructive pulmonary disease. These new models of care will be investigated more fully in patients with multimorbidity in the next phase of the programme.

### Moving forward

What is noteworthy about all of these innovative projects and programmes is that they were driven by the need for better care for patients with multimorbidity, by the need for optimization of systems and processes, and by the goal of achieving more effective, efficient, and satisfying services. Many of them have the potential to be up-scaled within their own countries or extended to and adopted by other regions and countries. In the future, the management and sustainability of these projects will be followed up and reviewed, and it is hoped that policymakers will utilize many of the ideas that have already been piloted and evaluated to devise powerful national solutions to the challenges posed by multimorbidity.

## Summary and conclusions

Patients with multimorbidity have complex and extensive medical needs that are not well served by current silo-based models of specialist care. Care is frequently fragmented, incomplete, and ineffective, and a lack of integration within healthcare systems and across care sectors can leave patients overwhelmed and unable to navigate their way towards better health outcomes. There are currently no Europe-wide and few national policies in place to address the need for more integrated care for people with multimorbidity, and this situation must be addressed as a matter of urgency. 

Many salient lessons can be learnt from the outstanding work undertaken at a European and national level in cancer and diabetes, and from the many excellent individual programmes and projects that have been undertaken at a regional and local level aimed at improving care for patients with multimorbidity. When developing care models for such patients, the focus should be on patient- (and people-) centred delivery, disease prevention, and health promotion, multidisciplinary teamwork, shared decision-making, sustainable financing, and on empowering the patient to self-manage. Multidisciplinary teams should include specialists, nurses, primary care physicians, pharmacists, physiotherapists, social care providers, and the patients, their families and carers, with all members of the team motivated, trained, and capable of delivering shared decision-making, common goals and objectives, and effective and sustainable self-management. Care pathways should be comprehensive, clear, accessible, flexible, and cost-effective. Ideally, patients should be provided with a single point of contact, a “care coordinator”, who helps them to navigate the healthcare system and access the right care at the right time. Care planning should be holistic, addressing the patient’s medical, psychosocial, and well-being needs, medication management issues, and self-care empowerment education. 

With the knowledge and expertise gained from work across all of the various sectors and organizations (medical, health, social, economic, political, educational, pharmaceutical, and non-governmental), developing effective, integrated models of care for patients with multimorbidity that could apply to every European country should now be within reach.

## Figures and Tables

**Table 1:** Examples of integrated care programmes addressing multimorbidity identified by the ICARE4EU (Innovating Care for People with Chronic Conditions in Europe) project (www.icare4eu.org).

Country	Programme	Main objectives	Target group	Key activities	Reference
Belgium	PROTOCOL 3 programme (ongoing)	To reduce the risk of institutionalization for the frail elderly	Community-dwelling frail elderly individuals (national programme)	Funding of innovative research projects aimed at providing alternative care models to support continued community dwelling	13
Bulgaria	Regional NPO providing diabetes care (ongoing)	To provide access to care for all patients with diabetes and other chronic illnesses	Patients with diabetes and their families, irrespective of age or insurance status (regional programme)	Care co-ordination across health service sectors, self-management skills, provision of primary care services, awareness campaigns – all delivered by volunteers	14
Cyprus	TeleRehabilitation programme (ongoing)	To apply advanced telemedicine to home rehabilitation monitoring	Patients discharged from ICU with multiple health issues (regional programme)	Home rehabilitation sessions with a physiotherapist connected via a video-communication system. Home monitoring of vital signs	15
Denmark	Clinic for Multimorbidity and Polypharmacy (ongoing)	To support primary care teams managing patients with multimorbidity	Patients with multimorbidity receiving polypharmacy (regional programme)	One-off comprehensive assessment by a multidisciplinary team of patients’ health and medication status, medication, with recommendations for follow-up	16
Finland	The POTKU project (Putting the Patient in the Driver’s Seat) (completed)	To improve patient-centred care for people with chronic illnesses and multimorbidity	People with a chronic condition seeking care from a local primary care centre – multimorbidity was highly prevalent (regional programme)	Development of personal health and care plans, assessment of self-management skills, development of integrated care services, and a care pathway for people with multimorbidity	17
Germany	The Gesundes Kinzigtal programme (ongoing)	To improve the health of the population, improve an individual’s experience of care, and reduce per capita costs	All individuals insured by two sickness funds in a rural area of South-West Germany (regional programme)	Self-management support, disease prevention, patient-centred care, and electronic networking system. Specific interventions for people with multimorbidity (e.g. polypharmacy reviews, disease prevention, and self-management training)	18
Spain	Strategy for chronic care in the Valencia region (ongoing)	To develop a comprehensive framework for an integrated model of care for patients with multimorbidity	Patients with complex medical needs and/or requiring palliative care (regional programme)	Joint collaborations between hospital and community nurse case managers to ensure continuity of care and mobilization of primary or secondary care resources as necessary. Stratification of patients according to morbidity profiles, drug therapy monitoring	19
The Netherlands	INCA model of integrated care for multimorbidity (ongoing)	To provide integrated care for patients with multimorbidity	Patients with multimorbidity (national programme)	Development of an integral modular approach to care built on existing standards of care, individualized care plans and risk profiling	20

ICU, intensive care unit; INCA, Integrated Care; NPO, non-profit organization.
