# Erratum to: Many diseases, one model of care?

**DOI:** 10.15256/joc.2016.6.78

**Published:** 2016-03-01

**Authors:** Tit Albreht, Mariana Dyakova, François G. Schellevis, Stephan Van den Broucke

**Affiliations:** ^1^Centre for Health System Analyses, National Institute of Public Health, Ljubljana, Slovenia; and CANCON Joint Action Programme, Ljubljana, Slovenia; ^2^Health Sciences, Warwick Medical School, University of Warwick, Coventry, UK; ^3^NIVEL (Netherlands Institute for Health Services Research), Utrecht, The Netherlands; and Department of General Practice and Elderly Care Medicine/EMGO Institute for Health and Care Research, VU University Medical Center, Amsterdam, The Netherlands; ^4^Psychological Sciences Research Institute, Université catholique de Louvain, Louvain-la-Neuve, Belgium

**Erratum to:** Albreht T, Dyakova M, Schellevis FG, Van den Broucke S. Many diseases, one model of care? J Comorbidity 2016;6(1):12–20. doi: 10.15256/joc.2016.6.73.

The received and accepted dates for this article were incorrectly published as Jan 4, 2015 and Jan 25, 2015, respectively, instead of Jan 4, 2016 and Jan 25, 2016, respectively. The original article has now been updated to reflect the correct submission and acceptance dates.

Journal of Comorbidity 2016;6(1):34

The original article can be found at: http://dx.doi.org/10.15256/joc.2016.6.73

